# Cross-Thinkers

**Published:** 1893-02

**Authors:** Kirke White


### CROSS-THINKERS.

For what end it may have been designed, we cannot tell; but the fact that in all questions, great and small, public and private, there is a class of minds which are sure to embrace the side of weakest argument. For a palpable and certain truth such persons have no relish. A great, broad principle, which recommends itself to the common sense of the bulk of mankind, is, in their eyes, an impertinence. In a doctrine everywhere prevalent and popular, they see only vulgarity.

A deduction irresistibly logical only excites in them the suspicion of some greater error.

If, on the other hand, you tell them something extremely hard to believe, they will make a manful struggle to swallow it, and probably will succeed. As Milton's Satan says, "Evil, be thou my good," so they cry "Sophism, be thou our reason."

The pious Jesuit who said "I believe it because it is impossible," was a type of this class. Any one can believe the possible,—there is no merit in them; but to accept in unshrinking faith something utterly incongruous with experience and common sense, is to do that which few can do, and to do it is, accordingly, great glory. There is some vanity in the matter after all. If I go with the multitude, my voice is lost in it. I may be right but I attract no attention. But if I stand up by myself, or with some small party or sect, and declare my attachment to some strangely heteroclite ideas, I at least do not pass noiselessly. The mass feel a little troubled by my dissent, and perhaps even think it worth while to take some pains to bring me over to their way of thinking. One becomes somebody in these circumstances.

It is also observable of this class of thinkers, that even when they concur with the majority in any profession of faith, they quite disregard all the leading and important points of the system and fasten exclusively upon some merely external or accidental peculiarities. A fundamental doctrine which most men feel goes down into the profoundest depths of their moral being, has no attraction for them; but they are careful to see the upholstery and millinery of the system preserved in all their ancient integrity. Just because a thing looks of no consequence they think it important. Were it really to become of consequence, they would desert it.

An almost superhuman suspiciousness is a constant feature of the cross-thinker. In his headlong tendency to suspect, he produces the most curious medley of ideas.

According to his way of thinking an author is not the author of his own books. There is always some person behind backs who writes them for him. He may write some other body's books, but not his own. When the cross-thinker sees a political opponent taking a course which shows a remarkable degree of moral courage, and obviously exposes him to damage in his worldly affairs, he feels assured there is some transcendental selfishness at the bottom of it. In considering by what means any great result has been brought about, our friends overlook all the prominent and great causes, and seldom fail, with an air of mysterious sagacity, to draw our attention to certain others so small, as to appear almost indifferent, or which possibly you are more inclined to rank as obstructions.

It is rather a sad reflection, that some of the men of most brilliant literary powers rank among those who devote themselves on all occasion, to make the worse appear the better reason. Unfortunately, to possess eloquence is not necessarily to possess also the inclination to use it solely for good ends. Crochet and vanity take the direction of but too much of it. The very fact that it is much easier to make a stir with eccentric opinions, than with those which have the support of truth and general approbation, is the cause why an immense proportion of the talent which arises from the first perverted, and ever afterwards misused. And we hardly know a more sad spectacle than that of a man of brilliant gifts being thus lead into false relations to his species, and condemned at the end to look back upon efforts of which the best that can be said is only this, that they have not been sufficiently powerful to extinguish truth, or obstruct the course of civilization.

Cross-thinking has a great charm for young minds. It is quaint and striking,—often droll, looks like something to which the Few are privileged, is free from that vulgarity which is so apt to beset any great cause in which the sympathies and interests of multitudes are concerned. Hence young men of talent are extremely liable to fall into the habit, and so get into connection with professions and parties from which they cannot afterwards shake themselves free. It is for them a great misfortune, for generally it tends to frustrate the benefits of what talent and education they may possess. Powers and accomplishments that might have advanced good objects for the public, are then spent in a necessarily futile attempt to obstruct them. Some false glory may result. In other words, a foolish few will applaud, while the majority look on with wonder and pity.

But in the long run, all is found to have been barren and wanting of true savor.

The world will, at the utmost, accord the meed of talents misapplied. Even from those who have all along been applauding, there will only be found that kind of support which the reckless get from their friends, and the vicious from the companions of their iniquity.

The final sentence is "Here lies a man who chose to live in vain."

KIRKE WHITE.

[We are inclined to the opinion that our new contributor in his cross-thinking essay aims at the free thinking propensity of one of our staff, now temporarily absent from his post of duty, who is heterodoxical enough to openly avow his disbelief that Jonah swallowed the whale, but with all his eccentricities of cross-thinking we have never heard him speak disparagingly of the whale as a fish, nor of Jonah as a prophet, as to any thing he knew for a fact beforehand.—Ed.]

